# Surface Modification of Sponge-like Porous Poly(3-hydroxybutyrate-*co*-4-hydroxybutyrate)/Gelatine Blend Scaffolds for Potential Biomedical Applications

**DOI:** 10.3390/polym14091710

**Published:** 2022-04-22

**Authors:** Mat Junoh Azuraini, Sevakumaran Vigneswari, Kai-Hee Huong, Wan M. Khairul, Abdul Khalil H.P.S., Seeram Ramakrishna, Al-Ashraf Abdullah Amirul

**Affiliations:** 1 School of Biological Sciences, Universiti Sains Malaysia, Penang 11800, Malaysia; azuraini1990@gmail.com (M.J.A.); kaihee.h.aa@m.titech.ac.jp (K.-H.H.); 2 Faculty of Science and Marine Environment, Universiti Malaysia Terengganu, Kuala Nerus 21030, Malaysia; vicky@umt.edu.my (S.V.); wmkhairul@umt.edu.my (W.M.K.); 3 Centre of Chemical Biology, Universiti Sains Malaysia, Penang 11900, Malaysia; 4 School of Life Science and Technology, Tokyo Institute of Technology, 4259 Nagatsuta, Midoriku, Yokohama 226-8501, Japan; 5 School of Industrial Technology, Universiti Sains Malaysia, Penang 11800, Malaysia; akhalilhps@gmail.com; 6 Department of Mechanical Engineering, Center for Nanotechnology and Sustainability, National University of Singapore, Singapore 119260, Singapore; seeram@nus.edu.sg; 7 Malaysian Institute of Pharmaceuticals and Nutraceuticals, NIBM, Penang 11700, Malaysia

**Keywords:** P(3HB-*co*-4HB), geazlatine, porous, freeze-drying, solvent casting, blend scaffolds

## Abstract

In this study, we described the preparation of sponge-like porous scaffolds that are feasible for medical applications. A porous structure provides a good microenvironment for cell attachment and proliferation. In this study, a biocompatible PHA, poly(3-hydroxybutyrate-co-4-hydroxybutyrate) was blended with gelatine to improve the copolymer’s hydrophilicity, while structural porosity was introduced into the scaffold via a combination of solvent casting and freeze-drying techniques. Scanning electron microscopy results revealed that the blended scaffolds exhibited higher porosity when the 4HB compositions of P(3HB-*co*-4HB) ranged from 27 mol% to 50 mol%, but porosity decreased with a high 4HB monomer composition of 82 mol%. The pore size, water absorption capacity, and cell proliferation assay results showed significant improvement after the final weight of blend scaffolds was reduced by half from the initial 0.79 g to 0.4 g. The pore size of 0.79g-(P27mol%G10) increased three-fold while the water absorption capacity of 0.4g-(P50mol%G10) increased to 325%. Meanwhile, the cell proliferation and attachment of 0.4g-(P50mol%G10) and 0.4g-(P82mol%G7.5) increased as compared to the initial seeding number. Based on the overall data obtained, we can conclude that the introduction of a small amount of gelatine into P(3HB-co-4HB) improved the physical and biological properties of blend scaffolds, and the 0.4g-(P50mol%G10) shows great potential for medical applications considering its unique structure and properties.

## 1. Introduction

Biomaterials have gained a lot of interest from researchers because they can aid in diseases or damaged cells in medical treatments [1,2]. These materials are designed to resemble native human tissue [3]. Biomaterials have been widely used for medical implants, such as sutures, bone plates, heart valves, as well as ligament and joint replacements [4,5]. In the early development of biomaterials, Dacron and stainless steel were used because they are relatively inert and tolerable to bodily responses. However, the drawback of these materials was that they were not biodegradable. Hence, biopolymers have been proposed as potential candidates to replace those materials because they are biodegradable and have good processability [1,3]. 

Polyhydroxyalkanoates are a group of polymers produced by microorganisms. They are completely biodegradable, biocompatible and exhibit non-genotoxicity for biomedical applications [6]. From the diverse range of PHAs, 4HB-copolymer has garnered attention in tissue engineering applications. In fact, the poly(3-hydroxybutyrate-*co*-4-hydroxybutyrate) copolymer was widely used as a material for medical devices, such as patches, sutures, tri-leaflet heart valves, bone scaffolds, and cardiac patches [7,8]. The poly(3-hydroxybutyrate-*co*-4-hydroxybutyrate) is a useful copolymer because its monomer unit possesses 3HB and 4HB units that are naturally found in human metabolites. Besides that, this copolymer has a wide variety of material properties ranging from a highly crystalline plastic to a strong elastomeric rubber-like material, depending on its 4HB monomer compositions (0–100) [9,10,11,12,13]. Besides that, the scaffolds fabricated from this copolymer exhibited degradation rate which can be controlled by manipulating its 4HB monomer composition [7]. Although it possesses many good properties, its main drawback is its low hydrophilicity, which hinders its functionality for tissue engineering applications. A scaffold should not only be able to biomimic the natural ECM but provide physical support for cells and contribute cell–surface interactions [14]. In this regard, scaffolds with improved hydrophilic functionalization are crucial. The presence of biomolecules, such as gelatine, fibronectin, laminin, or collagen, plays a key role in promoting cell–biomaterial interactions as the biological active sequences of the biomolecules improve cell adhesion, accelerate cell growth, and provide a favorable environment for their proper functioning [15].

Gelatine is a natural polymer that possesses a higher solubility and low antigenicity, hydrogel characteristics, biodegradability, and non-toxicity [16,17]. Furthermore, gelatine has excellent biocompatibility for cell attachment and proliferation because it contains the Arg-Gly-Asp (RGD)-like sequences of amino acids [18,19,20]. There have been many reports of gelatine blended with various polymers, such as polycaprolactone, poly(3-hydroxybutyrate), and poly(3-hydroxybutyrate-*co*-3-hydroxyvalerate). The incorporation of gelatine improves the hydrophilicity and promotes cell adhesion in these polymers. This is mainly targeted for medical applications, such as in dermal reconstruction and nerve tissue engineering in promoting cell–material interactions [21,22,23]. Table 1 lists common examples of biopolymers incorporated with gelatine to improve hydrophilic functionalization for various biomedical applications.

Various techniques have been used to fabricate porous scaffolds, such as salt leaching, gas foaming, solvent casting, and phase separation. However, some techniques, e.g., salt leaching and gas foaming, are quite tedious because they require additional procedures to remove the salt retained in the polymer matrix which may hinder the functionality of the scaffolds [5]. Hence, for convenience and ease on a smaller scale, solvent casting and freeze-drying were considered the best options. Freeze-drying is considered a versatile method to fabricate scaffolds with a three-dimensional (3D) porous structure with the removal of volatile organic residues, which is suitable for applications in drug delivery and bone tissue engineering [23].

In this study, we attempt to modify the surface morphology of a P(3HB-*co*-4HB) scaffolds by blending it with the natural polymer gelatine using a combination of solvent casting and freeze-drying techniques. The scaffolds were fabricated using various parameters, such as 4HB molar fraction, gelatine concentration, and final weight of the blend scaffolds, which resulted in a varying surface morphology. The study demonstrated that the varying surface morphology of P(3HB-*co*-4HB)/gelatine blend scaffolds with various physical and chemical properties can be fabricated with minor modifications of the processing parameters. The enhanced proliferation of fibroblast cells (L929) further indicates the potential application of the scaffolds in biomedical applications in the future.

## 2. Materials and Methods

### 2.1. Fabrication of P(3HB-co-4HB)/Gelatine Blend Scaffolds

Poly(3-hydroxybutyrate-co-4-hydroxybutyrate) [P(3HB-*co*-4HB)] with 4HB monomer compositions 27, 50, and 82 mol% with average molecular weights (Mw) of 1189, 736, and 434 kDa were synthesized using wild-type and transformant *Cupriavidus malaysiensis* USMAA 1020 [13,34]. The removal of endotoxins from the P(3HB-*co*-4HB) copolymer was carried out using hydrogen peroxide as previously described [35]. Gelatine from cold water fish skin (G7041, Bioreagent, solid), was purchased from Sigma Aldrich (St Louis, MO, USA).

**Figure 1:**
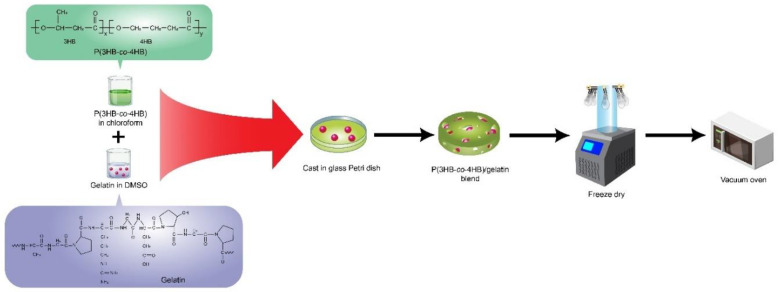
Schematic representation of the fabrication of P(3HB-*co*-4HB)/gelatine blend scaffolds which was prepared by solvent casting the mixture of fish gelatine and P(3HB-*co*-4HB) followed by freeze drying the scaffolds.

The P(3HB-*co*-4HB)/gelatine blend scaffolds were prepared using a combination of two different solvents: chloroform and dimethyl sulfoxide (DMSO). The blend scaffolds were prepared according to such parameters as the percentage of gelatin (7.5, 10 wt.%), 4HB monomer composition (27 mol%, 50 mol%, 82 mol%), and the final weight of blend scaffolds (0.79 g and 0.4 g). The P(3HB-*co*-4HB) copolymer was dissolved in chloroform (20 mL) while gelatine powder from cold fish skin (Sigma Aldrich, USA) was dissolved in DMSO (6 mL). Both solutions were stirred separately to ensure both polymers had completely dissolved. Then, the solutions were combined, stirred for 1 h, and carefully poured into the glass Petri dish (9 cm). The cast scaffold was washed with distilled water, freeze-dried (−75 °C) for 48 h, and later kept in a vacuum oven till further use, as shown in Figure 1 [36]. However, the highest gelatine concentration of only 7.5 wt.% could be incorporated to P(3HB-*co*-82mol%4HB) as a higher concentration disrupted the shape of the scaffolds. 

There were 6 scaffolds fabricated, which were thick and weighed 0.79 g, namely P(3HB-*co*-27mol%4HB)/10 wt.% gelatine 0.79g-(P27mol%G10); P(3HB-*co*-50mol%4HB)/10 wt.% gelatine 0.79g-(P50mol%G10); P(3HB-*co*-82mol%4HB)/10 wt.% gelatine 0.79g-(P82mol%G7.5) and as for those thin scaffolds weighing 0.4 g are P(3HB-*co*-27mol%4HB)/10 wt.% gelatine 0.4g-(P27mol%G10); P(3HB-*co*-50mol%4HB)/10 wt.% gelatine 0.4g-(P50mol%G10); P(3HB-*co*-82mol%4HB)/10 wt.% gelatine 0.4g-(P82mol%G7.5). The scaffolds will be known as listed above from here on.

### 2.2. Characterization of P(3HB-co-4HB)/Gelatine Blend Scaffolds

#### 2.2.1. Surface Morphology Analysis

Scanning electron microscopy (SEM) was used to study the surface morphology of the fabricated P(3HB-*co*-4HB) and P(3HB-*co*-4HB)/gelatine blend scaffolds. The samples (1 × 1 cm) were mounted on metal stubs and coated and viewed with a Leo Supra 50 VP Field Emission SEM (Carl-Zies SMT, Oberkochan, Germany) [37].

#### 2.2.2. Attenuated Total Reflection Fourier Transform Infrared Spectroscopy (ATR-FTIR) Analysis

An ATR-FTIR (Model RX1, PerkinElmer, Buckinghamshire, UK) was used to analyse the fabricated samples (P(3HB-*co*-4HB), gelatine, and P(3HB-*co*-4HB)/gelatine blend scaffolds) using FTIR spectroscopy. The IR spectra were obtained with wavelengths between 4000 and 400 cm^−1^ and analysed in transmittance mode [37].

#### 2.2.3. Pore Size and Porosity Analysis 

The pore size of the blend scaffolds was measured and calculated using image analyser software (Olympus Co. Ltd., Tokyo, Japan). A total of 30 different spots were analysed and averaged [26]. The porosity of blend scaffolds was determined from two SEM images for each group using the MATLAB software program [38]. According to the program code in MATLAB (R2019a), porosity was represented by the ratio of the total dark count spaces to the total pixels of the scaffold surface. The formula calculation of porosity was calculated as follows:

Porosity (%): (Total dark count/Total pixel) × 100

#### 2.2.4. Thickness Analysis

The image thickness of P(3HB-*co*-4HB) and P(3HB-*co*-4HB)/gelatine blend scaffolds was captured by an Olympus S240 Stereo Microscope (Olympus Co. Ltd., Tokyo, Japan) fitted with a JVC K-F55B (Kanagawa, Japan) colour video camera. The thickness of the blend scaffolds was measured and calculated using image analyser software (Olympus Co. Ltd., Tokyo, Japan). A total of six different spots were analysed and averaged.

#### 2.2.5. Atomic Force Microscopy (AFM)

The surface roughness of P(3HB-*co*-4HB) and P(3HB-*co*-4HB)/gelatine blend scaffolds were analyzed using AFM (Model Dimension Edge Bruker, Karlsruhe, Germany). The roughness (Rq), of the scaffold surface was calculated based on a standard formula integrated into the software. The sampling area was standardized at 10 × 10 µm. Five different spots per film were analyzed [37].

#### 2.2.6. Water Absorption Capacity

The water absorption capacity of blend scaffolds was determined by swelling the scaffolds in distilled water at room temperature. The scaffolds with recorded weights were placed in water for intervals of 5, 10, 15, 20, 25, and 30 min. The adsorbed water on the scaffold surface will be removed with filter paper and the wet weight of the scaffold will be measured immediately using an analytical balance. The percentage of water adsorption of the scaffolds was calculated according to

*E* = [*W_e_* − *W_o_*/*W_o_*] × 100 (1)

where *E* is the percentage of water adsorption at equilibrium, *W_e_* is the wet weight, and *W_o_* is the initial weight of the test scaffolds. Each swelling experiment was repeated three times and the average value taken as the percentage of water adsorption [39,40].

#### 2.2.7. Water Solubility of the Scaffold

The blend scaffolds were cut into small pieces (1 × 1 cm) and placed in a shake flask with 5 mL of distilled water. The samples were incubated in the oven at 37 °C for 72 h. Then, the scaffold pieces were dried overnight. The solubility of blend scaffolds was determined by measuring the decrease of weight of blend scaffolds after overnight drying. The percentage of solubility of blend scaffolds was calculated based on the formulation below:

Solubility (%) = ((*W_b_* − *W_a_*)/*W_b_*) × 100 (2)

where *W_a_* refers to the weight of scaffolds at the end of the experiment and *W_b_* denotes the weight of scaffolds at the beginning of the experiment. The average value for solubility in percentage was averaged from three replicates [37].

### 2.3. In Vitro Cell Culture

The in vitro cell culture was carried out using mouse fibroblast cell line (L929, ATCC). The cells were cultured in MEM (Minimal Essential Medium) supplemented with 2 mM l-glutamine, 1 mM sodium pyruvate, 1% minimum essential amino acid, 10,000 U/mL penicillin-streptomycin, and 10% of fetal bovine serum (FBS) and incubated at 37 °C in 5% CO_2_ for one to three days. Cell media were changed every 2 days and cells were passaged at ~80% confluency. The cytotoxicity of the fabricated P(3HB-*co*-4HB) and P(3HB-*co*-4HB)/gelatine blend scaffolds was tested by placing cut fabricated scaffolds discs with a diameter of 6 mm to fit a 96-well flat bottom culture plate. The 6 mm fabricated scaffolds were sterilized for one h prior to testing using GERMICIDE UV Sterilizer (CAMI, Parma, Italy). Cells were seeded at the concentration of 2.5 × 10^4^ cells/mL and incubated in a 5% CO_2_ incubator at 37 °C for 72 h. The cell viability and proliferation were assayed with MTS (3-(4,5-dimethylthiazol-2-yl)-5-(3-carboxymethoxyphenyl)-2-(4-sulfophenyl)-2H-tetrazolium/PMS (phenazine methosulfate) [8,35].

### 2.4. Statistical Analysis

Statistical analysis was performed using SPSS 16.0 software to conduct ANOVA and Tukey’s test. The significance level adopted was p-values more than 0.05 were considered significant. All results were presented with replication.

## 3. Results and Discussion

### 3.1. Surface Morphology and Roughness of P(3HB-co-4HB)/Gelatine Blend Scaffolds

**Figure 2:**
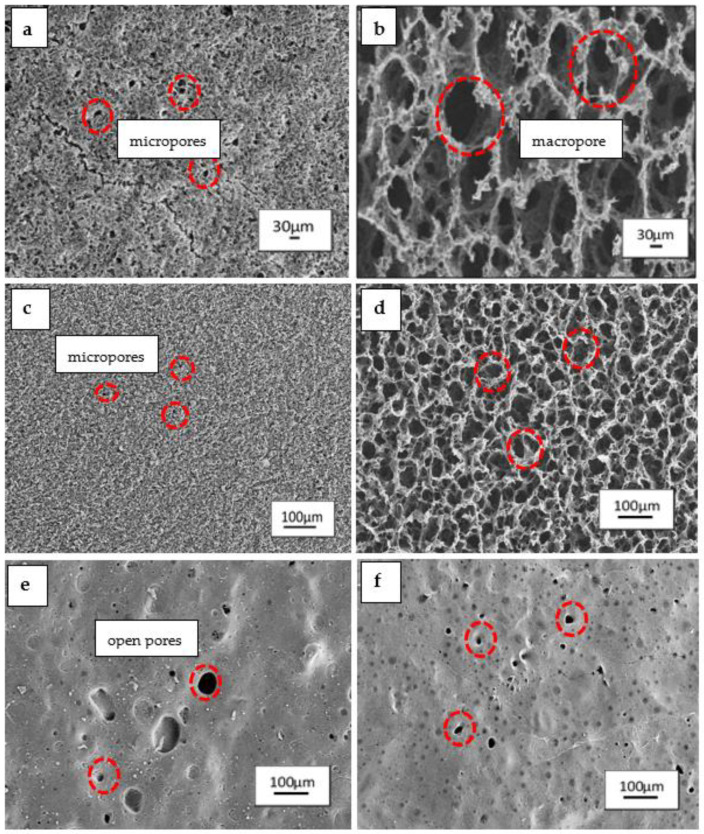
Surface morphology of P(3HB-co-4HB)/gelatine blend scaffolds with final weight of (**a**) 0.79g-(P27mol%G10); (**b**) 0.4g-(P27mol%G10); (**c**) 0.79g-(P50mol%G10); (**d**) 0.4g-(P50mol%G10); (**e**) 0.79g-(P82mol%G7.5); (**f**) 0.4g-(P82mol%G7.5).

The surface morphology of the scaffolds fabricated is crucial for tissue engineering. Scanning electron microscopy (SEM) was conducted to observe the microstructural changes and surface morphology of the P(3HB-*co*-4HB)/gelatine blend scaffolds fabricated using different 4HB monomer compositions and the final weight of blend scaffolds. Based on the Figure 2a,c, SEM revealed three-dimensional interconnected porous structure of the scaffolds 0.79g-(P27mol%G10) and 0.79g-(P50mol%G10) which possessed interconnected micropores structure which are compact and organised. As the final weight of the blends was reduced, the scaffolds of 0.4g-(P27mol%G10) and 0.4g-(P50mol%G10) demonstrated an open macrostructure with a higher degree of interconnectivity and porosity. However, the P(3HB-*co*-82mol% 4HB)/gelatine scaffold exhibited a smooth surface structure with open pores and a low degree of interconnectivity. A porous interconnected structure is favorable for cellular penetration, diffusion of nutrients, and degradation products of the scaffolds [41]. However, the range of pore size suitable for tissue engineering depends on the types of cells and the tissue to be engineered [42]. This was further proven with the pore size and porosity measurement (Table 2).

It was shown that the porosity of P(3HB-*co*-27mol% 4HB)/gelatine and P(3HB-*co*-50 mol% 4HB)/gelatine ranged from 16% to 35%. The porosity of both copolymers doubled, increasing from 20% to 40% after reducing the final weight of blend scaffolds. The pore size of P(3HB-*co*-27mol% 4HB)/gelatine blend scaffolds increased as the scaffolds weight was decreased. Meanwhile, the 0.79 g-(P50mol%G10) had the smallest pore size of 14 µm. Nevertheless, the reduction in weight to 0.4 g increased the pore size to 49 µm.

This could be attributed to the effect of freeze-drying process which may have enhanced pore inter-connectivity and networking [5]. Meanwhile, P(3HB-*co*-82mol% 4HB)/gelatine revealed a decreased pore size from 45 µm to 17 µm as the final weight of the scaffolds was reduced. The lowest porosity was also recorded with 0.79g-(P82mol%G7.5) and 0.4g-(P82mol%G7.5). The findings revealed that the manipulation of P(3HB-*co*-4HB) and final weight of blend scaffolds provided significant changes to the surface morphology, pore size, and porosity of blend scaffolds. Interestingly, the final weight reflects on the thickness of the scaffolds which is one of the key factors in scaffold design parameters [43]. Besides that, P(3HB-co-4HB) films changed from a hard crystalline to an elastic rubber texture when its 4HB monomer composition increased [12]. The polymer has a porous structure in crystalline state, and thus the porous structure reduced with the reduction of the crystalline structure within the polymer [12]. On the other hand, open interconnected porous structures of the scaffolds 0.79g-(P27mol%G10), 0.79g-(P50mol%G10), 0.4g-(P27mol%G10), and 0.4g-(P50mol%G10) were interesting as the micropores are advantageous as they provide anchorage sites for cell attachment and proliferation on the scaffolds. Basically, creating functionalized scaffolds from polymers that can be modified or tailored to mimic the extracellular matrix (ECM) has always been the key factor in fabricating scaffolds for biomedical applications [44,45,46].

### 3.2. FTIR Analysis of P(3HB-co-4HB)/Gelatine Blend Scaffolds

**Figure 3:**
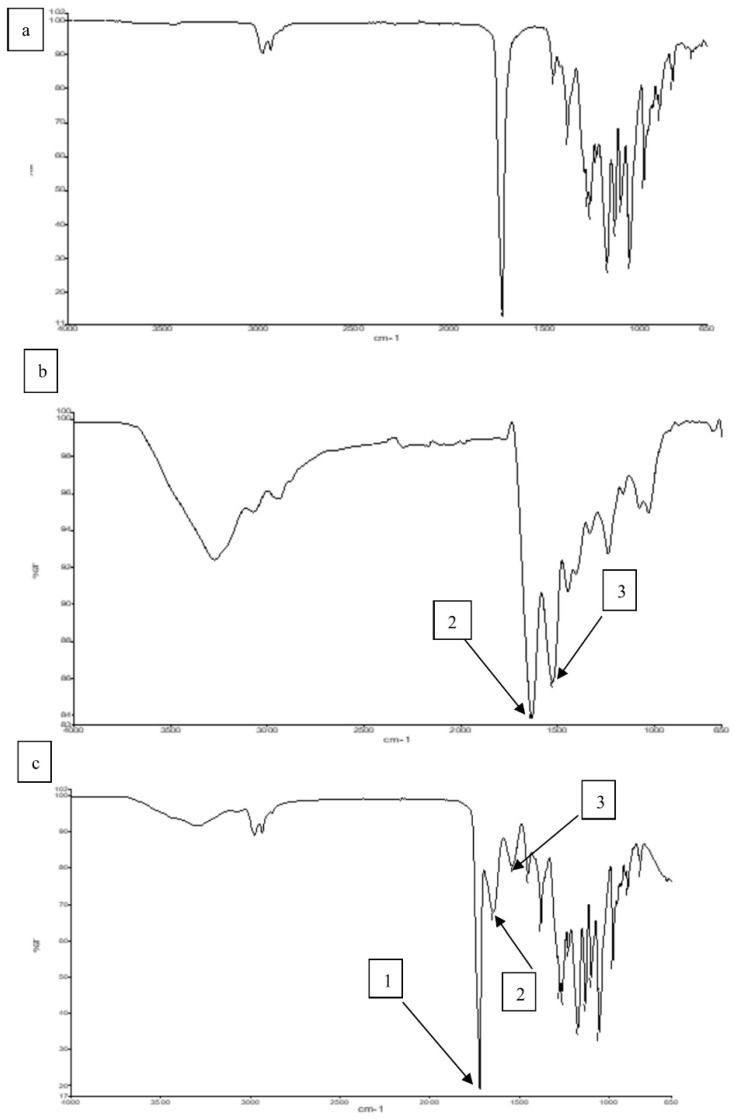
FTIR spectra of (**a**) P(3HB-*co*-4HB), (**b**) Gelatine (**c**) P(3HB-*co*-4HB)/gelatine blend scaffolds. Arrow 1, 2 and 3, indicate the ester group, amide I and amide II, respectively.

Figure 3a shows that the P(3HB-*co*-4HB) copolymer exhibited a prominent stretching of the carbonyl group (C=O) at 1720–1719 cm^−1^. For pure gelatine (Figure 3b), the peak at the 3250–3780 cm^−1^ region is represented by N-H bond stretching of hydrogen-bonded amide groups. Bands at 1660–1620 cm^−1^ denote the carbonyl C = O double bond stretching mode, with contributions from in-phase bending of the N–H bond and stretching of the C–N bond, referred to as amide I. Bands at 1550–1520 cm^−1^ referred as amide II, show deformation of the N–H bonds [20,47,48,49]. In Figure 3c, for the blend scaffold, the absorption bands of P(3HB-*co*-4HB) and gelatine were detected and there were also a number of bands overlapping each other which proves that the blending between both polymers has occurred. Besides that, the shifted band between P(3HB-*co*-4HB) and gelatine indicates that an intermolecular hydrogen bond formation between the carbonyl group of P(3HB-*co*-4HB) and the amide group of gelatine has taken place at band (~2500–2700 cm^−1^). A similar trend of this shifted band was found in a previous study [37,49], whereby the shifted band indicated an intermolecular hydrogen bond formation between P(3HB-*co*-4HB) and chitosan.

### 3.3. Effect of Thickness on Solubility and Water Absorption of P(3HB-Co-4HB)/Gelatine Blend Scaffolds

In tissue engineering, scaffolds fabricated should be stable and not leach out to achieve feasibility as cell culture matrices [50]. Table 3 shows the results of percentage of solubility of the various P(3HB-*co*-4HB)/gelatine blend scaffolds fabricated. The solubility of the blend scaffolds ranged from 2% to 16% and demonstrated the ability to retain more than 80% of their structure in a solution. The solubility of blend scaffolds decreased with an increase in the 4HB monomer composition from 27 mol% to 82 mol% for both final weights of the scaffolds of 0.79 g and 0.4 g. However, the 0.4g-P(3HB-*co*-4HB)/gelatine blend scaffolds posed the highest percentage of solubility ranging between 10% to 16%. This could be attributed to the thickness of the scaffolds. The thickness of the scaffolds has been reduced to half as the final weight of the scaffolds were reduced from 0.79 g to 0.4 g. The P(3HB-*co*-27mol% 4HB)/gelatine blend scaffolds were the thickest scaffolds with a thickness ranging from 0.96 mm to 1.81 mm. In contrast, both P(3HB-*co*-50 mol% 4HB)/gelatine and P(3HB-co-82mol% 4HB)/gelatine blend scaffolds had a reduction in thickness ranging from 0.50 mm to 0.32 mm and 0.44 mm to 0.22 mm respectively. Based on the solubility of the scaffolds, it can be deduced that scaffolds with higher final weight or thickness trap gelatine differently in the blend scaffolds. The thick scaffolds will have gelatine embedded in between the (3HB-*co*-4HB) copolymer thus, has low water solubility. Aside from that, the effect of intermolecular interaction, in this case between gelatine and P(3HB-*co*-4HB) copolymer, also influences the solubility of scaffolds [51,52]. Our results were in accordance with a previous work, where P(3HB-*co*-4HB)/chitosan exhibited good water resistance and low water solubility with an increase in 4HB monomer composition and chitosan content from 5 wt.% to 20 wt.% [37].

**Figure 4:**
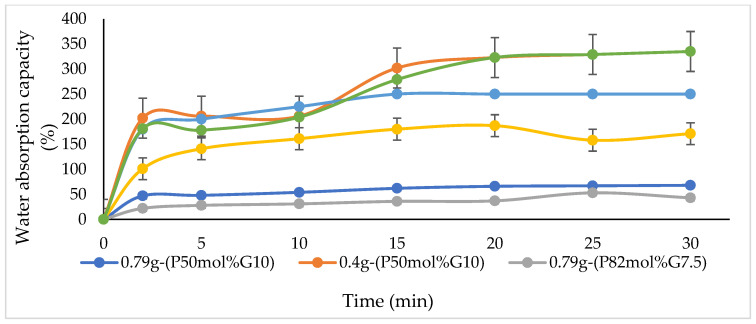
Water absorption capacity varying blend scaffolds.

Water absorption by materials indicates the hydrophilicity of the material in terms of the amount of water absorbed. Figure 4 demonstrates that the water absorption capacity of P(3HB-co-4HB)/gelatine blend scaffolds. Based on the results, the final water absorption is in the order of 0.4g-(P50mol%G10) > 0.4g-(P27mol%G10) > 0.79g-(P27mol%G10) > 0.4g-(P82mol%G7.5) > 0.79g-(P50mol%G10) > 0.79g-(P82mol%G7.5). The 0.4g-(P50mol%G10) showed the highest water absorption capacity with 325% as compared to the other scaffolds. Meanwhile, the improvement of water absorption capacity in this study can be correlated to the porosity of the blend scaffolds (Table 2) as previously described [37]. Gelatine will form a gel at low temperature and this allows for better absorbance of water [24,25,26,27,28,29]. Therefore, the hydrogel scaffold can absorb and retain water well. Hence, based on the results obtained, although P(3HB-co-4HB) is known to exhibit poor hydrophilicity, the scaffolds in this study have combined properties of both hydrogel and porous structures based on their high-water absorption capability. A similar observation was seen in a previous study where gelatine enhanced the swelling capacity of 250–260% in PLA/gelatine blend hydrogels deemed super absorbent [24]. This result suggested that 0.4 g-(P50mol%G10) was desirable as cell culture matrices for tissue engineering in the future [53].

### 3.4. In Vitro Proliferation of P(3HB-co-4HB)/Gelatine Blend Scaffolds

**Figure 5:**
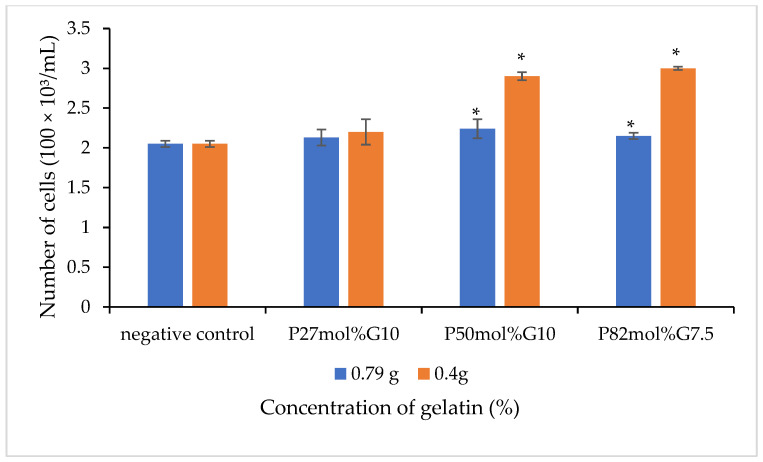
The cell viability of L929 cells of the various P(3HB-*co*-4HB)/gelatine blend scaffolds on day three for different contents of gelatin with final weights of 0.79 g and 0.4 g. Values are the mean of three replicates. Negative controls are P(3HB-*co*-4HB) without the incorporation of gelatine. Mean data accompanied by * asterisks represent a statistically significant difference within the group (Tukey’s HSD test, * *p* < 0.05).

The in vitro cell culture study was carried out to investigate the effect of blend scaffolds on the L929 murine fibroblasts cell line. The L929 cell line was seeded on the surface of scaffolds for 72 h and an MTS assay was carried out to determine the cell growth on the scaffolds. Figure 4 shows the number of cells that grew on the P(3HB-*co*-4HB)/gelatine blend scaffolds. Based on the results obtained in Figure 5, it is evident that the fabricated blend scaffolds did not exhibit any cytotoxicity and are able to support cell attachment and proliferation of L929 fibroblasts. From these results, the number of L929 fibroblasts cells increased as compared to the initial seeding. Based on the results, the highest cell proliferation was observed in 0.4 g-(P50mol%G10). The thickness of scaffolds greatly influenced the cell growth, whereby the thin scaffolds (those with final weight of 0.4 g) had a higher cell proliferation as compared to scaffolds fabricated with a final weight of 0.79 g. A similar trend is observed in a previous study, where the cell proliferation rate of P(3HB-*co*-4HB)/collagen peptide blend film showed that the L929 cell line grew better with a reduction of thickness from 0.2 mm to 0.1 mm [14]. The thickness of a scaffolds is crucial, especially in wound dressing, and porous collagen–chitosan scaffolds with a thickness of less than 1 mm were found to be ideal for dermal regeneration [54,55,56,57,58,59]. Apart from that, the 4HB monomer composition also influenced cell growth on scaffolds. The P(3HB-*co*-50 mol% 4HB) and P(3HB-*co*-82mol% 4HB) exhibited a higher number of cells compared to P(3HB-*co*-27mol% 4HB). It was apparent that the presence of gelatine, film thickness, and 4HB monomer composition enhanced cell proliferation and growth.

### 3.5. Surface Topography by Atomic Force Microscopy Analysis

**Figure 6:**
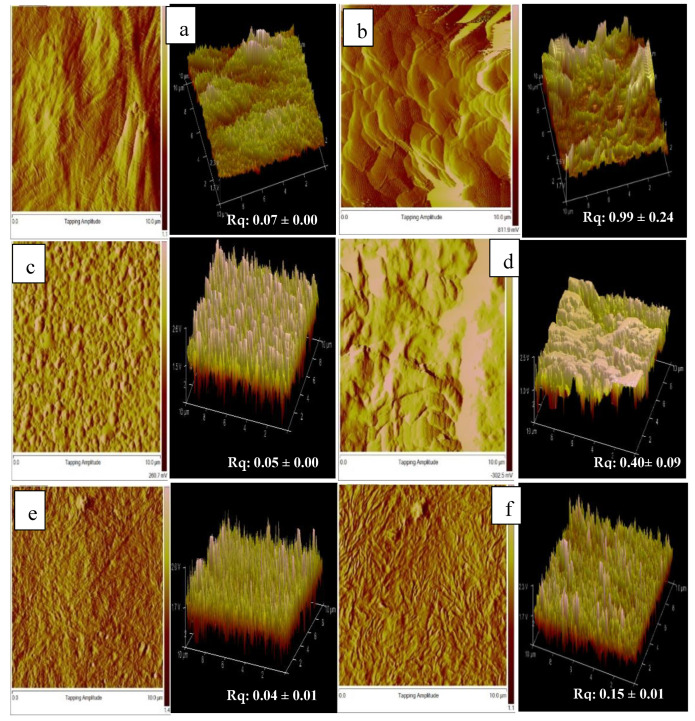
AFM topographic image of (**a**) 0.4g-(P27mo%), (**b**) 0.4g-(P27mol%G10); (**c**) 0.4g-(P50mol%), (**d**) 0.4g-(P50mol%G10); (**e**) 0.4g-(P82mo%) (**f**) 0.4g-(P82mol%G7.5).

The distribution of pores is fundamental in the fabrication of scaffolds to promote the formation of tissue. The findings obtained in this study revealed that the presence of gelatin gelatine, film thickness and different 4HB monomer compositions significantly influenced the surface roughness topography of blend scaffolds. However, it has been proven that scaffolds with final weight of 0.4 g enhanced the proliferation and growth of fibroblast cells. Figure 6 shows the atomic force microscopy of the surface roughness of 0.4g-P(3HB-*co*-4HB)/gelatine blend scaffolds. The mean roughness of the root mean square of the z values, Rq ranged from 0.04 µm to 0.99 µm. The results implied the P(3HB-*co*-4HB) films have a smooth surface structure. The surface roughness of the blend scaffolds decreased in the following order: 0.4g-(P27mol%G10) > 0.4g-(P50mol%G10) > 0.4g-(P82mol%G10). The 0.4g-(P50mol%G10) and 0.4g-(P82mol%G10) had rougher surfaces which explains the higher cell proliferation and attachment in these scaffolds. As previously described by Sumerneva et al. [60], the surface topography influences surface wettability. The surface topography with rougher surfaces has higher surface wettability due to the large surface area of porous surface [61]. The advantage of surface roughness is the increase of the interaction between the cell and scaffold, which is useful for nutrient absorption and the removal of metabolites through the pores [62].

## 4. Conclusions

In this study, we demonstrated that P(3HB-*co*-4HB)/gelatine blend scaffolds exhibit various interesting properties due to the incorporation of gelatine, such as providing a spongy-like structure. This increases their ability to absorb and retain water, resulting in a high-water absorption for 0.4g-(P50mol%G10). The incorporation of the biomolecule gelatine improved the hydrophilicity of the polymer, thus increasing the cell attachment. Hence, the 0.4g-(P50mol%G10) scaffold could be an excellent biomaterial to be potentially developed for tissue engineering applications upon further investigations.

## Figures and Tables

**Table 1:** List of common examples materials incorporated with gelatine to improve surface functionalisation.

Biopolymer/ Materials	Types of Cells	Applications	References
Poly(lactic-*co*-glycolic) acid (PLGA)	Human Mesenchymal Stem (hMSC)	Myocardial tissue engineering	[24]
Poly(lactic-*co*-glycolic) acid (PLGA)	Human umbilical vein endothelial cells (HUVECs)	Soft tissue engineering applications	[25]
Poly(l-lactic acid) (PLLA)	Neonatal human dermal fibroblasts (NHDF)	Skin tissue engineering	[26]
P(3HB-*co*-4HB)/PVA	Human Bone Marrow Mesenchymal Stem Cells (hBMSCs)	Scaffold for tissue engineering	[27]
Polycaprolactone (PCL), nanohydroxyapatite (nHAp).	Human osteoblast cell line	Bone tissue engineering	[28]
Poly(3-hydroxybutyrate-*co*-3-hydroxyvalerate) (PHBV)	3T3 fibroblasts, HaCat keratinocytes	Diabetic wound healing	[29]
Gellan gum	Human dermal fibroblast (HDF), bone marrow-derived mesenchymal stem cells (hMSC) and adipose-derived stem cells (ADSC)	Burn wound therapy	[30]
Poly(3-hydroxybutyrate-*co*-4-hydroxybutyrate) (P(3HB-*co*-4HB))/pullulan	Schwan cells (RSC96)	Drug delivery applications.	[31]
Pullulan	Osteoblast precursor cell line (MC3T3)	Bio-hydrogel for biomedical applications	[32]
Gellan gum	Human-induced pluripotent stem cells (hiPSC)-derived cardiomycytes	Cardiac tissue engineering	[33]

**Table 2:** The pore size and porosity of P(3HB-*co*-4HB)/gelatine blend scaffolds at different final weight of blend scaffolds.

Scaffold formulation	Pore size (µm)	Porosity (%)
0.79g-(P27mol%G10)	21 ± 4	20 ± 0
0.79g-(P50mol%G10)	14 ± 3	21 ± 3
0.79g-(P82mol%G7.5)	45 ± 16	5 ± 0
0.4g-(P27mol%G10)	64 ± 21	40 ± 1
0.4g-(P50mol%G10)	49 ± 9	40 ± 0
0.4g-(P82mol%G7.5)	17 ± 7	5 ± 3

**Table 3:** The thickness and solubility of P(3HB-*co*-4HB)/gelatine blend scaffolds.

Scaffolds	Thickness (mm)	Solubility (%)	Retain (%)
0.79g-(P27mol%G10)	1.81 ± 0.064	12 ± 2	88 ± 2
0.79g-(P50mol%G10)	0.50 ± 0.015	3 ± 0	97 ± 0
0.79g-(P82mol%G7.5)	0.44 ± 0.014	2 ± 1	98 ± 1
0.4g-(P27mol%G10)	0.96 ± 0.014	16 ± 3	84 ± 3
0.4g-(P50mol%G10)	0.32 ± 0.012	12 ± 1	88 ± 1
0.4g-(P82mol%G7.5)	0.22 ± 0.015	10 ± 1	90 ± 1

Values are means ± SD of three replicates. Means in the same column that are labeled with different alphabets are significantly.different (Tukey’s HSD test, *p* < 0.05).
